# Exercise and Productive Hippocampal Neurogenesis After Stroke: From Niche Remodeling to Cognitive Recovery

**DOI:** 10.3390/brainsci16090951

**Published:** 2026-09-07

**Authors:** Xinzheng Wang, Guohao Yi, Huifen Zhou, Bihan Wang, Qiang Tu

**Affiliations:** 1 Faculty of Health Sciences and Physical Education, Macao Polytechnic University, Macao 999078, China; 2 Department of Physical Education, Zhejiang Chinese Medical University, Hangzhou 310053, China; 3 School of Basic Medical Sciences, Zhejiang Chinese Medical University, Hangzhou 310053, China; 20121021@zcmu.edu.cn; 4 School of Physical Education, Hunan Normal University, Changsha 410012, China; 5 Faculty of Medicine and Health, The University of Sydney, Sydney 2006, Australia; qiang.tu@sydney.edu.au

**Keywords:** post-stroke cognitive impairment, exercise, adult hippocampal neurogenesis, neuroinflammation, neurovascular unit, mitochondrial homeostasis

## Abstract

**Highlights:**

**What are the main findings?**

- Exercise may support post-stroke cognitive recovery through coordinated regulation of neuroinflammation, neurovascular repair, and mitochondrial homeostasis within the hippocampal niche.
- Productive adult hippocampal neurogenesis requires newborn neuron survival, maturation, and functional integration, rather than progenitor proliferation alone; however, its causal contribution to human cognitive recovery remains unresolved.

**What are the implications of the main findings?**

- The niche-permissiveness framework provides a mechanistic model linking exercise interventions with hippocampal remodeling and productive neurogenesis after stroke.
- Future studies combining lineage tracing, targeted neurogenesis manipulation, multimodal biomarkers, and domain-specific cognitive assessments are needed to establish causality and enhance clinical translation.

**Abstract:**

Post-stroke cognitive impairment (PSCI) is a frequent and disabling consequence of stroke that limits long-term recovery and quality of life. Disruption of the hippocampal neurogenic niche may impair adult hippocampal neurogenesis (AHN), while exercise is increasingly recognized as a promising non-pharmacological strategy for cognitive rehabilitation. This review synthesizes evidence that exercise may shift the post-stroke hippocampal niche from a hostile to a permissive state through coordinated regulation of neuroinflammation, neurovascular integrity, and mitochondrial homeostasis. We distinguish increased progenitor proliferation from productive neurogenesis, defined by the survival, maturation, appropriate positioning, and functional integration of newborn neurons. Exercise may attenuate inflammatory signaling, support angiogenesis and blood–brain barrier repair, and improve mitochondrial biogenesis and quality control; however, much of the mechanistic evidence remains preclinical, and increases in early neurogenic markers do not by themselves establish functional neuronal integration or causality. Current clinical evidence supports cognitive benefits of exercise more strongly than it supports AHN as the indispensable mediator of those benefits. A productive-neurogenesis framework therefore provides a more rigorous basis for interpreting existing studies and designing future experiments that combine lineage tracing, temporally controlled neurogenesis ablation, circuit-level analysis, and domain-specific cognitive outcomes.

## 1. Introduction

Stroke remains a leading cause of death and disability worldwide, and the global number of stroke-related deaths is projected to increase substantially between 2020 and 2050 [1,2]. Post-stroke cognitive impairment (PSCI) commonly affects learning, memory, attention, and executive function, thereby limiting independent living and long-term recovery [3]. Available pharmacological treatments mainly target functional, language, or emotional symptoms and may produce adverse effects, while their effects on pathological brain remodeling and sustained cognitive recovery remain limited [4,5,6,7]. Adult hippocampal neurogenesis (AHN) contributes to hippocampus-dependent learning and memory and may participate in structural and functional recovery after stroke [8,9].

Primary experimental studies suggest that exercise may increase neurogenesis-related markers, enhance neurotrophic and neurovascular signaling, and improve hippocampus-dependent cognition after cerebral ischemia [9,10,11]. Exercise-regulated inflammatory and muscle–brain signaling pathways have also been implicated in post-stroke neurogenesis and cognitive recovery [12,13]. However, exercise should not be regarded as a uniform intervention [14]. Treadmill running and voluntary wheel running differ in controllability and voluntariness and may produce distinct neuroplastic and cognitive effects [15]. Whole-body vibration and exercise preconditioning provide different physiological stimuli and are applied at different stages relative to cerebral ischemia [16,17]. Resistance exercise may influence hippocampal plasticity, but direct evidence linking it to post-stroke AHN remains limited [18]. Accordingly, the most direct evidence linking exercise to post-stroke AHN currently derives from aerobic animal paradigms. Furthermore, few studies have simultaneously assessed niche remodeling, newborn-neuron maturation, functional integration, and cognition within a single causal design.

Targeted searches of PubMed, the Cochrane Library, Embase, Web of Science, CNKI, and WanFang Data were conducted to identify literature available online or in print up to 28 February 2026. Search terms covered exercise, physical training, stroke, cerebral ischemia, cognition, hippocampal neurogenesis, neuroinflammation, neurovascular function, and mitochondrial homeostasis. Priority was given to primary preclinical and clinical studies that examined exercise in stroke or related cerebral ischemic conditions and reported hippocampal, mechanistic, neurogenic, or cognitive outcomes. Relevant reviews and non-stroke studies were used to define broader concepts or supplement pathways for which stroke-specific evidence was limited. Importantly, increased expression of proliferation-related or immature-neuron markers does not necessarily demonstrate productive neurogenesis, which requires the survival, maturation, and functional integration of newborn neurons. Accordingly, this review distinguishes evidence of progenitor proliferation from evidence of productive neurogenesis and evaluates the causal and translational boundaries of the proposed mechanism. This mechanistic narrative review integrates these findings to clarify how exercise may remodel the post-stroke hippocampal niche and inform the development of mechanism-based exercise interventions.

## 2. Pathophysiological Basis of Post-Stroke Cognitive Impairment and AHN

Following stroke, the central nervous system undergoes a series of acute and chronic pathophysiological changes that contribute to cognitive impairment. Post-stroke cognitive impairment (PSCI) generally develops within 3 to 6 months after stroke onset. Studies indicate that approximately 50–70% of stroke survivors experience symptoms of PSCI [19,20,21]. PSCI encompasses not only deficits specific to the lesion location, such as aphasia and memory impairment, but also dysfunction resulting from infarction in the hippocampus, thalamus, and other critical cortical regions. These regions are closely associated with adult hippocampal neurogenesis (AHN). AHN refers to the process by which neural stem/progenitor cells in the subgranular zone (SGZ) of the dentate gyrus continuously proliferate and differentiate into new granule cells. This process constitutes a crucial cellular basis of brain plasticity and is essential for the maintenance of cognitive functions such as learning and memory, as well as for post-injury repair [14]. Stroke-induced hippocampal injury is closely associated with the onset and progression of cognitive impairment and represents a major pathological link in post-stroke cognitive dysfunction [22]. Multiple studies have shown that stroke-triggered factors, including cerebrovascular injury [23], neuroinflammation, and mitochondrial dysfunction [24], jointly disrupt the structure and function of the hippocampus and other brain regions, thereby suppressing AHN. Reduced AHN is closely associated with cognitive decline after stroke and may represent an important cellular basis for this impairment. The following sections discuss the effects of stroke on AHN from three perspectives.

### 2.1. Neuroinflammation and Adult Hippocampal Neurogenesis After Stroke

Cerebral ischemia rapidly disrupts energy metabolism and induces cellular injury. Necrotic cells release damage-associated molecular patterns (DAMPs), such as high mobility group box 1 (HMGB1), which rapidly activate resident immune cells, predominantly microglia. This activation initiates inflammatory transcriptional programs involving nuclear factor kappa B (NF-κB), mitogen-activated protein kinase (MAPK), and related pathways. As a result, pro-inflammatory cytokines, including interleukin-1β (IL-1β), tumor necrosis factor-α (TNF-α), and interleukin-6 (IL-6), are upregulated, thereby amplifying inflammation and inducing structural and functional neuronal damage [25,26]. These events constitute an important pathophysiological basis for secondary brain injury and post-stroke functional impairment.

A large body of evidence indicates that neuroinflammation exerts a significant inhibitory effect on neurogenesis [27,28]. TNF-α, IL-1β, and IL-6 are among the most prominently elevated pro-inflammatory cytokines after stroke. Their signaling can directly affect neural stem cells (NSCs), thereby altering proliferation and differentiation [29,30]. These cytokines may suppress the proliferative capacity of NSCs through activation of NF-κB-related inflammatory pathways and are associated with reduced Ki67 expression and fewer BrdU-labeled cells [16]. In addition, p38 mitogen-activated protein kinase (p38 MAPK) is activated after stroke and is associated with both pro-inflammatory cytokine upregulation and cellular injury [31].

Clinical studies have reported significantly increased levels of inflammatory mediators, including IL-6 and neutrophils, in the plasma and cerebrospinal fluid during the acute phase of stroke [32]. Excessive inflammation not only causes secondary damage to surviving neurons but also inhibits neural stem cells and newly generated neurons. In animal models, persistent inflammation significantly reduces the number of newborn neurons in the subgranular zone (SGZ) of the hippocampus, thereby suppressing neurogenesis [33]. Kathner-Schaffert et al. [34] assessed neuronal status and learning and memory performance in middle cerebral artery occlusion (MCAO) mice using the Morris water maze (MWM) and immunohistochemical markers. They found that all MCAO groups exhibited impaired MWM performance. Early ischemic injury led to a marked age-related decline in neurogenesis, which was associated with the premature development of hippocampus-dependent deficits. This study supports the view that stroke may lead to long-term cognitive impairment.

**Figure 1:**
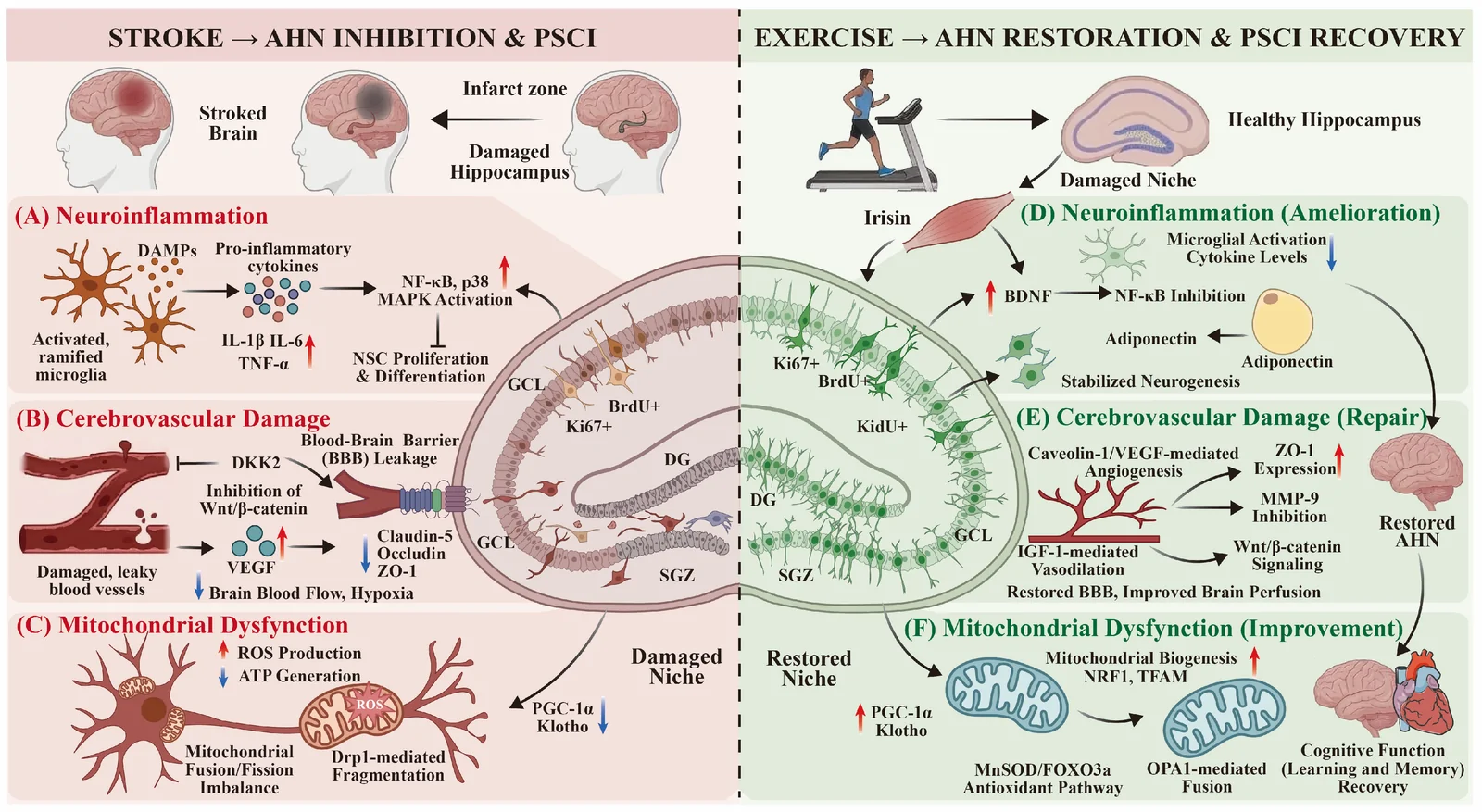
Proposed mechanisms through which exercise may restore a permissive hippocampal neurogenic niche after stroke. (**A**) Post-stroke neuroinflammation; (**B**) cerebrovascular injury and blood–brain barrier dysfunction; (**C**) mitochondrial dysfunction; (**D**) exercise-associated attenuation of neuroinflammation; (**E**) exercise-associated repair of neurovascular injury; and (**F**) exercise-associated restoration of mitochondrial homeostasis. Through coordinated regulation of these processes, exercise may support the survival, maturation, and integration of newborn neurons. AHN, adult hippocampal neurogenesis; BBB, blood–brain barrier; BDNF, brain-derived neurotrophic factor. The red upward arrow indicated an increase, while the blue downward arrow represented a decrease in content.

Taken together, post-stroke hippocampal neuroinflammation is closely associated with impaired adult hippocampal neurogenesis. Elevated inflammatory signaling in the hippocampus suppresses neurogenesis and contributes to cognitive dysfunction (Figure 1A).

### 2.2. Cerebrovascular Injury and Adult Hippocampal Neurogenesis After Stroke

Cerebrovascular injury represents a key pathological feature of ischemic stroke. Following ischemia, cerebral microvascular endothelial cells develop metabolic disturbances and oxidative stress. These changes impair tight junction proteins, including claudin-5, occludin, and ZO-1, thereby increasing the paracellular permeability of the blood–brain barrier (BBB) [35]. In the early stage of cerebral ischemia, ischemia-induced upregulation of vascular endothelial growth factor (VEGF) further increases BBB permeability, thereby exacerbating cerebral edema and increasing the risk of hemorrhagic transformation [36].

Cerebrovascular injury directly disrupts the microenvironment of plastic brain regions such as the hippocampus. Reduced blood and oxygen supply limits the nutrients and energy required for neural stem cell (NSC) proliferation and newborn neuron survival. Post-stroke cerebrovascular occlusion is a major trigger for disruption of the vascular niche that supports adult hippocampal neurogenesis (AHN). The dense microvascular network in the hippocampus provides essential structural support for neurogenesis. After stroke, cerebral blood flow and microvascular density in the hippocampus decline significantly. As a result, insufficient oxygen and nutrient supply markedly impair NSC proliferative capacity and shorten the survival of newborn neurons [37]. In addition, structural and functional vascular impairment may indirectly suppress AHN [38].

Moreover, blood–brain barrier disruption is another important mechanism through which post-stroke cerebrovascular injury impairs AHN. Recent studies have shown that Dickkopf-related protein 2 (DKK2), which is abundantly secreted by ischemic neurons, aggravates BBB disruption by inhibiting the Wnt/β-catenin signaling pathway, which is essential for cell survival and vascular stability, thereby contributing to neuronal injury [39]. Transcriptomic analyses have further shown that reactivation of the PI3K/CREB pathway can stimulate adult neurogenesis in the presence of stroke-related microvascular dysfunction [40]. Chen et al. [10] demonstrated that exercise or pharmacological intervention promoted cerebrovascular repair after stroke and significantly restored hippocampal microvascular density and BBB integrity. These changes reversed AHN suppression and improved cognitive function, indirectly supporting the crucial regulatory role of the cerebrovascular niche in AHN.

Taken together, post-stroke cerebrovascular dysfunction plays a critical role in central nervous system dysregulation and impaired hippocampal AHN. Ameliorating post-stroke vascular damage and BBB disruption may help restore AHN and promote cognitive recovery (Figure 1B).

### 2.3. Mitochondrial Dysfunction and Adult Hippocampal Neurogenesis After Stroke

Mitochondrial dysfunction is a prominent feature of the post-stroke brain. It is characterized by reduced mitochondrial biogenesis, excessive production of reactive oxygen species (ROS), and impaired mitochondrial quality control, including fusion, fission, and mitophagy. These interconnected defects exacerbate neuronal injury and death [41].

During post-stroke ischemia–reperfusion, excessive ROS generation is a key contributor to mitochondrial dysfunction [42]. ROS disrupts mitochondrial structural integrity, reduces oxidative phosphorylation efficiency, and limits adenosine triphosphate (ATP) production [43]. As a result, the proliferation and differentiation of neural stem cells (NSCs) are impaired [44]. Excessive ROS accumulation and disrupted Ca^2+^ homeostasis can induce opening of the mitochondrial permeability transition pore (mPTP) and loss of membrane potential, further impairing ATP production and promoting cell death. Sustained ROS accumulation can further activate apoptotic signaling pathways and promote neuronal apoptosis [45]. Studies have shown that ROS levels in the hippocampus are significantly elevated after stroke, whereas the activities of antioxidant enzymes such as glutathione peroxidase (GPx) and superoxide dismutase 2 (SOD2) are reduced. These changes are accompanied by increased malondialdehyde (MDA) levels. Enhancing antioxidant enzyme expression to alleviate mitochondrial oxidative damage can significantly promote AHN [43,45].

In addition to oxidative injury, reduced mitochondrial biogenesis and impaired quality control further aggravate AHN suppression after stroke [46]. Mitochondrial biogenesis is largely regulated by peroxisome proliferator-activated receptor gamma (PPAR-γ) and its coactivator, peroxisome proliferator-activated receptor gamma coactivator 1-alpha (PGC-1α). After stroke, activation of the Ca^2+^/CREB/PGC-1α signaling pathway is impaired, resulting in reduced mitochondrial abundance and function. This deficit fails to meet the energy demands of NSC proliferation and differentiation [44].

Meanwhile, disruption of mitochondrial quality control mechanisms, including fusion, fission, and mitophagy, prevents defective mitochondria from being effectively eliminated, thereby aggravating oxidative stress and disordered energy metabolism [47]. In particular, imbalance in the PINK1/Parkin-mediated mitophagy pathway and autophagy-lysosomal flux promotes the retention of damaged mitochondria and sustained ROS production, thereby creating a vicious cycle. After stroke, Klotho expression is downregulated and the FOXO3a/MnSOD antioxidant axis is impaired, resulting in reduced mitochondrial ROS clearance. This change is detrimental to the survival and differentiation of neural progenitor cells [48]. In contrast, exercise-related irisin treatment can increase Klotho expression and ameliorate cognitive impairment after cerebral ischemia.

Collectively, these mechanisms lead to widespread mitochondrial dysfunction in the post-stroke hippocampus, which represents an important intrinsic factor underlying AHN suppression. Taken together, stroke suppresses AHN and contributes to cognitive decline through multiple mitochondrial pathways, including reduced biogenesis and impaired quality control processes such as fusion, fission, and mitophagy (Figure 1C).

## 3. Potential Mechanisms Underlying the Effects of Exercise on Post-Stroke Neurogenesis and Cognitive Function

AHN may contribute to cognitive recovery, whereas impaired AHN may be involved in post-stroke cognitive dysfunction. As a non-pharmacological intervention, exercise promotes post-stroke cognitive recovery through multiple targets and pathways. These effects may involve remodeling of the hippocampal neurogenic niche, attenuation of pathological injury, and support of neurogenesis-related processes [10,49]. Based on recent advances in this field, we discuss the potential molecular mechanisms through which exercise alleviates post-stroke AHN impairment and cognitive decline (Figure 1).

### 3.1. Exercise Attenuates Post-Stroke Neuroinflammation, Promotes AHN, and Improves Cognitive Function

Stroke induces inflammatory responses in the hippocampus, thereby impairing neurogenesis and cognitive function. Exercise can ameliorate these adverse effects [50]. These effects involve multiple mechanisms, including suppression of inflammatory signaling pathways and enhancement of anti-inflammatory factor production. Specifically, exercise can directly inhibit pro-inflammatory signaling pathways, thereby counteracting the inhibitory effects of inflammatory mediators on hippocampal neurogenesis. Fan et al. [29] further supported these beneficial effects of exercise. In that study, rats with chronic cerebral hypoperfusion (CCH) underwent 4 weeks of voluntary wheel running. Exercise significantly reduced the levels of pro-inflammatory cytokines, including IL-1β, TNF-α, and IL-6, through the TLR4/MyD88/p38 MAPK signaling pathway. It also reduced hippocampal neuronal loss and improved cognitive performance, providing valuable insight into the prevention and treatment of post-stroke cognitive impairment. In the inflammatory cascade, NF-κB is a key transcription factor that regulates the expression of multiple inflammatory genes and may crosstalk with neurotrophic signaling pathways such as brain-derived neurotrophic factor (BDNF). NF-κB may also promote synaptogenesis through glutamate-related mechanisms [51]. Conversely, inhibition of NF-κB suppresses neuroinflammation and protects neuronal cells [52]. Based on RNA sequencing, Song et al. [12] showed that sedentary stroke rats exhibited upregulation of inflammation-related genes, including NF-κB, along with poorer learning and memory performance. In contrast, exercise exerted anti-inflammatory effects by suppressing the expression of NF-κB and other inflammation-related genes, thereby stabilizing neurogenesis and significantly improving cognition.

In addition to central inflammatory signaling, exercise may modulate peripheral inflammation and metabolic status, thereby reducing the inflammatory burden in the brain. For example, in ischemia-related models, exercise reduces the direct damage caused by inflammatory cells to the central nervous system, thereby improving serum cytokine profiles and cognitive function. These effects may be related to exercise-induced reductions in TNF-α and IL-1β levels, which inhibit β-cell apoptosis and increase β-cell mass [31].

Furthermore, exercise-induced enhancement of post-stroke hippocampal neurogenesis is associated with increased levels of myokines and neurotrophic factors. Irisin, a muscle-derived hormone, may regulate brain metabolism and inflammation and appears to play an important role in the exercise-mediated attenuation of post-stroke neuroinflammation and promotion of neurogenesis. Thirty days of low-frequency whole-body vibration (WBV) training significantly reduced circulating levels of pro-inflammatory cytokines, including IL-1β, IL-6, IL-18, IFN-γ, and TNF-α, in rats after transient middle cerebral artery occlusion (tMCAO). It also increased serum irisin and BDNF levels, supporting the efficacy of exercise in improving cognitive deficits [16]. Consistently, Wei et al. [13] reported that the FNDC5/irisin/BDNF axis was downregulated in both stroke patients and multiple animal models. Enhancement of this axis through exercise or peripheral irisin administration increased hippocampal BDNF levels and the abundance of immature neurons, thereby improving PSCI. In line with these findings, hippocampal BDNF is associated with neuronal survival, synaptic plasticity, and memory performance [53]. BDNF-TrkB signaling mediates hippocampal neurogenesis, and activation of this pathway is closely associated with exercise-induced improvement in hippocampus-dependent memory [54]. Hong et al. [11] reported that 4 weeks of treadmill exercise increased hippocampal BDNF and TrkB expression in stroke mice, enhanced synaptic plasticity and neurogenesis, and improved motor function and short-term memory.

In addition, adiponectin, a metabolic factor secreted by adipose tissue, contributes to the regulation of AHN after stroke. Reduced adiponectin levels after stroke are associated with suppressed AHN, whereas exercise-induced increases in adiponectin can reduce hippocampal neuronal apoptosis and promote newborn neuron survival through activation of the AdipoR1/AMPK pathway [17]. Taken together, exercise attenuates post-stroke hippocampal inflammation through multiple pathways, promotes AHN, and improves cognitive functions such as learning and memory (Figure 1D).

### 3.2. Exercise Attenuates Post-Stroke Cerebrovascular Injury, Promotes AHN, and Improves Cognitive Function

Exercise can enhance post-stroke hippocampal remodeling and improve learning and memory by promoting angiogenesis, restoring blood–brain barrier integrity, and improving cerebral perfusion. Chen et al. [10] provided further support for this view. In their study, 2 weeks of treadmill exercise significantly upregulated caveolin-1, BDNF, and VEGF expression in the ischemic penumbra of middle cerebral artery occlusion (MCAO) mice. Exercise also promoted endothelial cell proliferation through the Caveolin-1/VEGF pathway, increased capillary density around the infarct area, as indicated by elevated BrdU/CD34-positive cells, and enhanced angiogenesis, neurogenesis, and synaptic plasticity. These changes ultimately ameliorated post-stroke motor and cognitive impairments.

A randomized clinical trial showed that an 8-week combined cardiorespiratory and resistance exercise program after ischemic stroke was safe and improved an executive-function measure at 12 months, but did not preserve hippocampal volume relative to balance and stretching [55]. Exercise can also activate the PI3K/Akt/eNOS pathway via insulin-like growth factor 1 (IGF-1), increase the expression and phosphorylation of endothelial nitric oxide synthase (eNOS), enhance vasodilatory function, and improve cerebral blood flow [37,56,57]. These effects help create a stable microenvironment for adult hippocampal neurogenesis (AHN) and ultimately support cognitive recovery. After stroke, excessive activation of matrix metalloproteinase-9 (MMP-9) disrupts blood–brain barrier tight junction proteins and aggravates hippocampal microvascular injury. Exercise significantly inhibits MMP-9 activity and upregulates zonula occludens-1 (ZO-1) expression, thereby reducing hippocampal microvascular damage and limiting the entry of peripheral inflammatory factors into the hippocampus, where they may interfere with AHN [38].

Long-term exercise increases BDNF expression, promotes post-ischemic myelin repair and microvascular remodeling, and helps reconstruct an intact neurovascular unit (NVU), thereby facilitating recovery after injury [58]. Four weeks of treadmill exercise improved spatial memory and increased hippocampal BrdU-, DCX-, and NeuN-related neurogenesis measures, accompanied by restoration of BDNF–pCREB signaling [59]. These coordinated reparative effects on the vasculature and nervous system provide an important structural and microenvironmental basis for exercise-mediated cognitive recovery after stroke (Figure 1E).

### 3.3. Exercise Attenuates Post-Stroke Mitochondrial Dysfunction, Promotes AHN, and Improves Cognitive Function

After stroke, ROS levels increase markedly, triggering mitochondrial dysfunction in hippocampal neural cells and ultimately contributing to cognitive decline. Exercise is a well-established non-pharmacological intervention that improves mitochondrial function and plays a pivotal role in attenuating stroke-induced mitochondrial dysfunction [60,61].

First, exercise reduces mitochondrial oxidative stress by activating antioxidant signaling pathways. Excessive ROS production in the hippocampus after stroke disrupts mitochondrial structure and function. In contrast, exercise-induced irisin significantly upregulates antioxidant-related proteins in MCAO mice, including Klotho, manganese superoxide dismutase (MnSOD), and forkhead box O3a (FOXO3a). By modulating the Klotho pathway, irisin enhances mitochondrial ROS clearance and ameliorates cognitive impairment after cerebral ischemia [48]. Karizmeh et al. [43] reported that exercise preconditioning increased hippocampal Klotho expression, promoted MnSOD transcription, reduced ROS levels, protected mitochondrial function in neural cells, and preserved their proliferative and differentiative capacity, thereby improving working memory. Exercise also maintains the activity of antioxidant systems such as glutathione (GSH) in the hippocampus and prefrontal cortex, thereby further alleviating mitochondrial oxidative damage and protecting AHN [45].

In addition, exercise promotes mitochondrial biogenesis through pathways involving Ca^2+^ signaling, cAMP response element-binding protein (CREB), and peroxisome proliferator-activated receptor gamma coactivator-1 alpha (PGC-1α), thereby improving post-stroke energy supply. Exercise has been reported to upregulate key regulators such as PGC-1α, nuclear respiratory factor 1 (NRF1), and mitochondrial transcription factor A (TFAM), thereby increasing mitochondrial abundance and functional capacity in neurons and neural stem cells after stroke [41,62]. These changes help meet the energy demands of AHN. Exercise-associated increases in BDNF may support neuronal survival, plasticity, and neurogenesis-related processes after cerebral ischemia [60]. This process increases mitochondrial abundance and oxidative phosphorylation efficiency, thereby providing sufficient ATP for the maturation of newborn neurons [44].

Exercise training can enhance neural function by regulating mitochondrial quality control. Mitochondrial sirtuin 3 (SIRT3) is also critical for maintaining mitochondrial homeostasis [63]. After stroke, imbalance in mitochondrial fusion, fission, and mitophagy prevents defective mitochondria from being effectively removed. In contrast, exercise can inhibit excessive mitochondrial fission by regulating dynamin-related protein 1 (Drp1) while simultaneously promoting mitophagy and maintaining mitochondrial network homeostasis [41]. Previous studies have suggested that exercise training improves cognitive function by modulating mitochondrial dynamics and activating autophagy in aging hippocampal neurons [64]. Zhang et al. [65] reported that 2 weeks of exercise preconditioning increased the level of optic atrophy 1 (OPA1), a mitochondrial fusion regulator, in the ischemic cortex of young male rats, thereby reducing cerebral edema and improving neurological function. Qin et al. [66] further showed that exercise preconditioning increased the levels of the mitophagy-related proteins Bnip3L and Parkin in the ischemic cortex of MCAO mice. It also upregulated the mitochondrial fusion regulator Mfn2 (mitofusin 2) and the mitochondrial fission regulator Drp1, thereby ameliorating neurological dysfunction after ischemic stroke. This comprehensive regulation of mitochondrial function provides stable energy support for multiple stages of AHN and thereby contributes to cognitive recovery after stroke (Figure 1F).

These mechanisms are closely interconnected rather than independent. Post-ischemic inflammatory responses may impair endothelial function and blood–brain barrier integrity, whereas barrier disruption can facilitate the entry of peripheral immune cells and mediators, further amplifying neuroinflammation [67]. Neurovascular insufficiency may also restrict oxygen and substrate delivery, thereby intensifying mitochondrial energetic stress and ROS production. Conversely, mitochondrial dysfunction in cerebrovascular endothelial cells can directly increase blood–brain barrier permeability [68]. Disrupted mitochondrial quality control may further promote neuroglial activation, blood–brain barrier injury, and neuroinflammation, creating a self-reinforcing cycle within the post-stroke neurovascular niche [69]. Exercise may therefore support AHN by coordinately attenuating inflammatory signaling, promoting vascular repair, and improving mitochondrial homeostasis rather than by acting through a single isolated pathway. However, few studies have examined these mechanisms simultaneously, and their temporal and causal relationships remain to be established.

### 3.4. Exercise Modality, Dose, and Timing

Exercise modality, dose, and timing vary substantially across the available evidence (Table 1). Treadmill running is the most frequently studied post-stroke paradigm and provides the most controllable training exposure. Voluntary wheel running reduces coercive stress but produces less standardized exercise doses. In focal cerebral ischemia, voluntary running enhanced newborn-cell survival and spatial-memory recovery, whereas forced swimming produced no comparable benefit [9]. Whole-body vibration also improved hippocampus-dependent cognition after tMCAO [16], but it is not physiologically equivalent to active running. Exercise preconditioning engages several neuroprotective pathways [17,23,43,65,66], yet it primarily informs ischemic tolerance rather than post-stroke rehabilitation. These distinctions indicate that effects observed with one paradigm should not be generalized to all forms of exercise.

Direct comparisons between aerobic and resistance exercise in stroke-related AHN remain limited. In a non-stroke animal study, sustained aerobic running increased AHN, whereas resistance training did not alter proliferation, maturation, or survival markers of newborn neurons [70]. Recent randomized studies suggest that resistance training may influence hippocampal structure and related biomarkers in older adults and individuals with mild cognitive impairment [18,71]. However, these findings do not establish an effect on post-stroke AHN. Aerobic exercise therefore has more direct experimental support in post-stroke models, although current evidence is insufficient to determine whether aerobic or resistance exercise is superior for productive neurogenesis.

Clinical evidence remains preliminary. In the PISCES-ZODIAC trial, combined aerobic and resistance training after ischemic stroke was safe and improved executive performance but did not preserve hippocampal volume [55]. Broader clinical evidence supports exercise for brain health while also showing that improved fitness does not necessarily translate into cognitive benefit [72,73]. Moreover, hippocampal volume, circulating biomarkers, and cognitive performance are not direct measures of AHN. Interpretation is further constrained by small samples, heterogeneous protocols, incomplete reporting of randomization and blinding, limited representation of both sexes, and reliance on proliferation- or immature-neuron markers. Future post-stroke trials should compare clearly defined exercise modalities and doses using domain-specific cognitive outcomes together with imaging and molecular indicators.

**Table 1:** Preclinical and direct clinical evidence for exercise-related cognitive, hippocampal, and neurogenesis outcomes after stroke or cerebral ischemia.

Study	Population/Sample	Ischemia Model	Exercise Modality	Intensity, Duration, Frequency and Timing	Principal Outcomes
Preclinical evidence					
Seo et al., 2014 [60]	Gerbils, 8 weeks; *n* = 40 (10/group)	5 min bilateral carotid occlusion	Treadmill running	2–8 m/min, 30 min/day for 2 weeks; post-ischemia	Improved memory; increased BrdU/DCX and BDNF; reduced apoptosis.
Ahn et al., 2016 [58]	Aged male gerbils, 22–24 months; *n* = 63	5 min bilateral carotid occlusion	Treadmill running	5–10 m/min, 30 min/day, 5 days/week for 1 or 4 weeks; started day 5	Four weeks improved memory, neurogenesis-related measures, myelin/microvessels and BDNF.
Choi et al., 2016 [59]	Male Wistar rats, 8 weeks; *n* = 52	Bilateral carotid occlusion (2VO)	Treadmill running	15 m/min, 30 min/day, 5 days/week for 4 weeks; started week 3	Improved spatial memory and BrdU/DCX/NeuN measures; restored BDNF–pCREB signaling.
Stradecki-Cohan et al., 2017 [49]	Male rats, 3 months; focal *n* = 41; global *n* = 27	90 min MCAO or 8 min cardiac arrest	Graded treadmill running	30 min/day for 5–6 days; started after 3–4 recovery days	Moderate exercise produced the most consistent contextual-memory benefit.
Zhao et al., 2017 [74]	Male rats, 250–300 g; *n* = 80	Four-vessel occlusion	Treadmill running	20 m/min for 30 min versus progressive running to exhaustion; post-ischemia	Moderate protocol favored memory and neuronal survival; exhaustive exercise was detrimental.
Pan et al., 2017 [75]	Male hypertensive rats, 10–12 weeks; *n* = 90	90 min tMCAO	Motorized running wheel	20 min twice daily for 26 days, ~3–6 m/min; started day 3	Improved recognition memory and neuronal-proliferation/synaptic-plasticity markers.
Chen et al., 2019 [10]	Male C57BL/6 mice; *n* = 204	MCAO	Treadmill running	8–10 m/min, 30 min/day, 5 days/week for 7 or 14 days; started at 24 h	Improved spatial learning and caveolin-1/VEGF-, BDNF-, angiogenesis- and neurogenesis-related outcomes.
Hong et al., 2020 [11]	Male C57BL/6 mice, 8 weeks; *n* = 40	Photothrombotic stroke	Treadmill running	3–8 m/min, 30 min/day for 28 days; started day 1	Improved memory; increased synaptic proteins, BrdU/DCX cells and BDNF/TrkB signaling.
Pan et al., 2021 [76]	Juvenile male rats; *n* = 90 (18/group)	90 min tMCAO	Low-, moderate- or high-intensity treadmill	5, 10 or 15 m/min, 30 min/day, days 2–14 after stroke	Moderate intensity best supported spatial cognition and hippocampal synaptic outcomes.
Kerr et al., 2022 [16]	Middle-aged rats of both sexes; *n* = 112	90 min tMCAO	Whole-body vibration	~40 Hz, 15 min twice daily, 5 days/week for 30 days; started day 1	Improved cognition in both sexes; reduced inflammatory mediators and increased irisin/BDNF.
Karizmeh et al., 2022 [43]	Male rats, 250–300 g; *n* = 135	MCAO	Treadmill preconditioning	20 m/min, 30 min/day, 6 days/week for 3 weeks before MCAO	Improved memory and neurological outcomes; associated with Klotho/MnSOD signaling.
Song et al., 2024 [12]	Male rats, 10 weeks; *n* = 36	Photothrombotic infarction	Graded treadmill running	18, 24 or 30 m/min, 5 days/week for 4 weeks post-stroke; session duration/start NR	Low/moderate intensities favored memory and neurogenesis-related profiles over high intensity.
Zheng et al., 2025 [17]	Male C57BL/6J mice, 5–6 weeks; main experiment *n* = 7/group	MCAO	Treadmill preconditioning	10–12 m/min, 1 h/day for 14 days; MCAO 24 h after final session	Reduced infarction, gliosis and memory deficits; benefits involved adiponectin signaling.
Direct clinical evidence					
Ademoyegun et al., 2025 [32]	Ischemic stroke; 50 randomized, 48 completed; mean age 64.2 years; 27 men/21 women; baseline NIHSS 7.13.	First mild–moderate ischemic stroke	Very early multicomponent exercise	45 min twice daily for 7 days; initiated within 24 h; subsequent physiotherapy for 3 months	Improved inflammatory, functional and MoCA outcomes versus usual care.
Brodtmann et al., 2025 [55]	Ischemic stroke; 107 randomized, 104 analyzed; mean age 64 years; 67 men; median mRS 1 and NIHSS 0.	Ischemic stroke	Aerobic plus resistance exercise	Three 60 min sessions/week for 8 weeks; commenced ~2 months after stroke	Safe; improved executive performance but did not preserve hippocampal volume.

Abbreviations: BDNF, brain-derived neurotrophic factor; DCX, doublecortin; MCAO, middle cerebral artery occlusion; tMCAO, transient MCAO.

### 3.5. Evidence Strength and Causal Boundaries of Exercise-Induced Neurogenesis

Although many experimental studies report that exercise increases proliferation- and neurogenesis-associated markers, their biological interpretation requires caution. Ki67, BrdU/EdU, and doublecortin represent different stages of the neurogenic process and, when used individually, cannot establish the long-term survival, neuronal identity, appropriate migration, or functional integration of newborn cells. This distinction is particularly important after stroke, because ischemia may increase progenitor proliferation while disrupting the maturation and integration of newly generated neurons. Experimental stroke has been associated with more newborn dentate granule cells but also with ectopic localization, aberrant morphology, and impaired hippocampus-dependent memory [77]. The therapeutic objective should therefore be productive neurogenesis rather than simply maximizing the number of cells expressing an early neurogenic marker.

The causal contribution of AHN to exercise-mediated cognitive recovery is also incompletely established. Conditional ablation of neural progenitor cells impaired post-stroke spatial learning and reduced connectivity between the dentate gyrus and entorhinal cortex, supporting a functional contribution of endogenous neurogenesis to cognitive recovery [78]. However, this observation does not establish AHN as the principal mediator of exercise. Exercise simultaneously modifies cerebral perfusion, synaptic plasticity, neurotrophic signaling, systemic metabolism, sleep, mood, and cardiorespiratory fitness. Studies combining exercise with temporally controlled inhibition of neurogenesis are needed to determine whether its cognitive benefits are abolished, attenuated, or preserved.

Translation to patients requires additional caution. The persistence and magnitude of AHN in adult humans have long been debated because post-mortem interval, tissue fixation, antigen retrieval, disease status, and marker specificity can influence the detection of immature neurons [79,80,81]. Recent multiomic single-cell analyses have identified neural stem cells, neuroblasts, and immature granule neurons in the adult human hippocampus, providing molecular support for persistent AHN [82]. Nevertheless, these approaches provide cross-sectional molecular evidence and cannot directly establish the lineage origin, long-term survival, appropriate positioning, synaptic integration, or functional contribution of putative newborn neurons. Moreover, post-mortem studies cannot longitudinally track the maturation of individual cells or determine whether they become functionally incorporated into hippocampal circuits. Hippocampal volume, circulating BDNF, inflammatory mediators, and cognitive scores are complementary translational indicators, but none constitutes a direct in vivo measure of AHN. Current human evidence therefore supports the persistence of AHN and the cognitive benefits of exercise more strongly than it supports productive AHN as a causal or indispensable mediator of post-stroke cognitive recovery.

## 4. Summary and Future Perspectives

Centered on adult hippocampal neurogenesis (AHN), this review integrates evidence concerning the mechanisms through which exercise may regulate neuroinflammation, the neurovascular unit, and mitochondrial homeostasis after stroke. This framework provides an integrative perspective for investigating exercise-based interventions for post-stroke cognitive impairment.

Although acute recanalization therapy and secondary prevention strategies have continued to improve [83], several limitations constrain the interpretation of evidence linking exercise to AHN and cognitive recovery. First, substantial heterogeneity exists in exercise modality, intensity, duration, frequency, and timing [7], as well as in stroke and chronic cerebral hypoperfusion models [84] and cognitive assessments. This heterogeneity limits direct comparisons among studies and the identification of optimal exercise prescriptions. Second, the current evidence remains dominated by animal studies, whereas direct evidence in patients is limited. Third, many studies rely on proliferation or immature-neuron markers without establishing the long-term survival, maturation, appropriate positioning, or functional integration of newborn neurons. Fourth, few studies have directly manipulated AHN during exercise interventions, and its causal contribution to exercise-mediated cognitive recovery therefore remains uncertain. Finally, the persistence, magnitude, and functional relevance of AHN in adult humans remain incompletely resolved.

These limitations indicate several priorities for future research. First, exercise studies should directly compare modality, intensity, duration, frequency, and treatment timing across standardized experimental models. Second, lineage tracing [85] should be combined with mature-neuron markers, morphological assessment, and circuit-level analyses to determine whether exercise-induced newborn neurons survive and become functionally integrated. Temporally controlled inhibition or ablation of neurogenesis during exercise interventions would further help determine whether AHN is necessary for cognitive improvement. Third, studies should examine the interactions among neuroinflammatory, neurovascular, and mitochondrial pathways rather than investigating them only as parallel mechanisms. Finally, clinical research should integrate domain-specific cognitive assessments with multimodal indicators, including hippocampal perfusion and volumetric imaging, peripheral myokines, inflammatory profiles, and brain-derived neurotrophic factor. These indicators should be interpreted as complementary measures of hippocampal niche remodeling rather than direct in vivo evidence of AHN.

We propose a niche-permissiveness model in which neuroinflammation, neurovascular dysfunction, and mitochondrial impairment form an interacting set of constraints that determine whether progenitor activation progresses toward productive neurogenesis. Persistent inflammatory signaling may impair endothelial support and mitochondrial homeostasis, vascular insufficiency may restrict oxygen and trophic-factor delivery, and mitochondrial dysfunction may amplify oxidative and inflammatory signaling. Exercise may therefore support AHN not through a single pathway but by shifting the injured hippocampal niche toward a state in which newborn neurons can survive, mature, and integrate.

## Data Availability

No new data were created or analyzed in this study. Data sharing is not applicable to this article.

## Abbreviations

AHN	Adult hippocampal neurogenesis
BBB	Blood–brain barrier
BDNF	Brain-derived neurotrophic factor
MCAO	Middle cerebral artery occlusion
NSC	Neural stem cell
PSCI	Post-stroke cognitive impairment
ROS	Reactive oxygen species
SGZ	Subgranular zone
